# Thymic Hyperplasia-Associated Autoimmune Encephalitis Mimicking Neuroleptic Malignant Syndrome: A Case Report

**DOI:** 10.7759/cureus.31186

**Published:** 2022-11-07

**Authors:** Mohammad Abu-Abaa, Halyna Pylypiv, Ali Abdulsahib, Doaa Ali

**Affiliations:** 1 Internal Medicine, Capital Health Regional Medical Center, Trenton, USA

**Keywords:** new episode psychosis, sympathetic storming, neuroleptic malignant syndrome (nms), anti-nmdar encephalitis, thymus

## Abstract

Anti-NMDA (N-methyl-D-aspartate) receptor encephalitis is a common autoimmune encephalitis. It is commonly associated with underlying malignancy. We present a 24-year-old patient with sudden onset of behavioral changes and acute psychosis who was treated with antipsychotics followed by the development of generalized rigidity, facial twitching, and sympathetic overactivity. MRI and EEG were inconclusive. The neuroleptic malignant syndrome was presumed and bromocriptine was started. Multiple antiepileptics were started to control this twitching without success. NMDA receptor reactivity in the CSF established the diagnosis. Searching for underlying malignancy was unyielding except for an anterior mediastinal mass seen on the CT chest. The patient had only mild improvement in response to corticosteroids, plasmapheresis, and intravenous immunoglobulin (IVIG). Significant improvement was achieved after the thymectomy. This case serves to remind clinicians of key aspects of the disease including general rigidity and paroxysmal sympathetic hyperactivity, and the potential to confuse these with other diagnoses including neuroleptic malignant syndrome. This case is also unique in that the association with thymic hyperplasia is very rare and only a few cases were reported in English literature.

## Introduction

Anti-NMDA (N-methyl-D-aspartate) receptor encephalitis is a relatively new entity, identified around 15 years ago. The main features of clinical presentation include sudden psychiatric symptoms or cognitive impairment, and seizures. Pathology involves antibodies that are directed against the glutamate receptor NMDA. It is quite challenging to identify this disease at the time of onset. It is more prevalent in women. Many tumors have been identified in association with the disease. This case serves to remind clinicians of key aspects of the disease including general rigidity and paroxysmal sympathetic hyperactivity, and the potential to confuse these with other diagnoses including neuroleptic malignant syndrome. This case is also unique in that the association with thymic hyperplasia is very rare and only a few cases were reported in English literature.

## Case presentation

**Figure 1:**
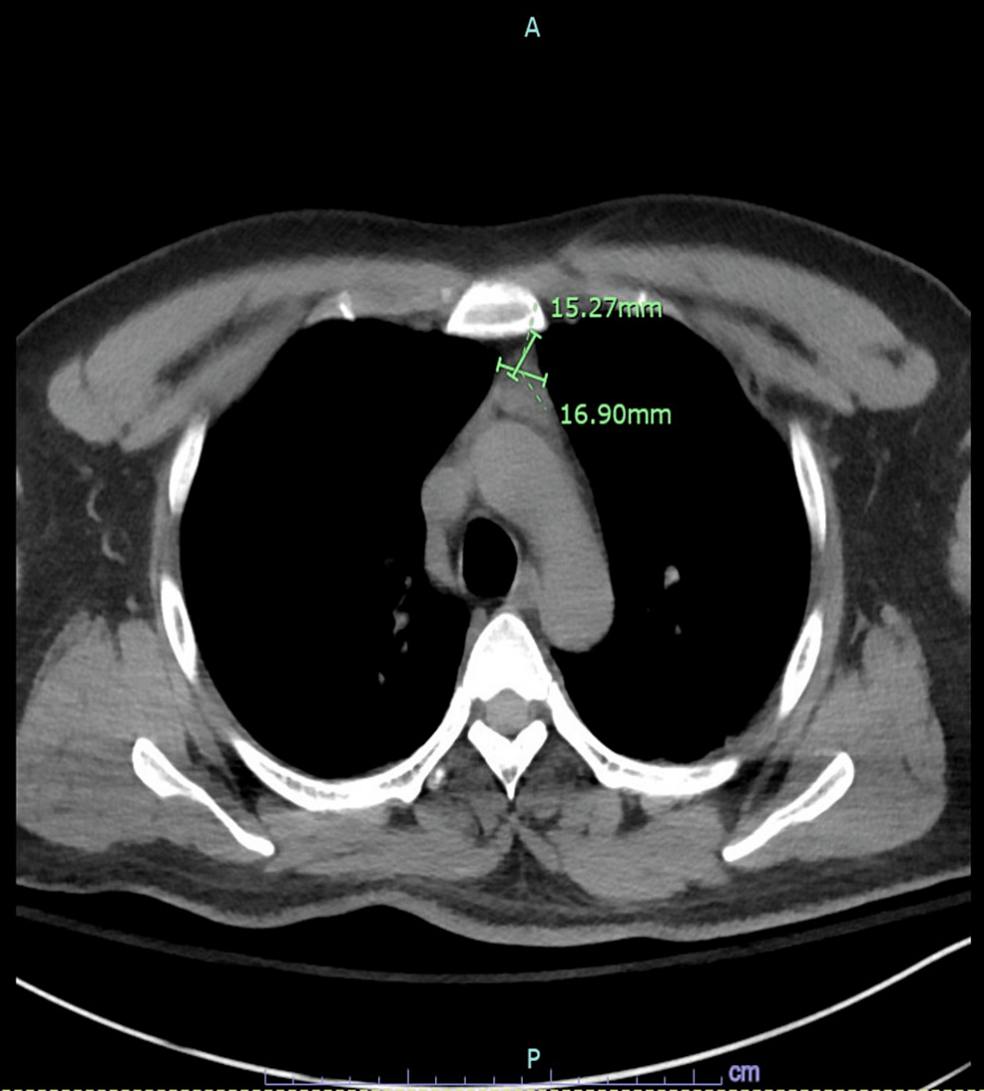
Thymic mass Computed tomography of the chest showing anterior mediastinal mass.

A 24-year-old male with no past medical history presented after breaking into a stranger’s car and refusing to leave. He mentioned having visual and auditory hallucinations. Collateral history included the recent new onset of bizarre behavior and hyperreligiosity. Information collected from relatives was unremarkable for past medical, family, and psycho-social history. On exam, he was combative, responding to internal stimuli, and non-cooperative. Initial labs showed only reactive leukocytosis and hyperCKemia with a negative toxicology screen. Agitation was managed by haloperidol around the clock. Magnetic resonance imaging (MRI) brain was unyielding. Computed tomography (CT) of the chest and abdomen were unremarkable except for soft tissue density in the anterior mediastinum (Figure 1).

Cerebrospinal fluid (CSF) analysis was unremarkable. The CSF was negative for cryptococcal, Epstein Barr virus (EBV), cytomegalovirus (CMV), and venereal disease research lab (VDRL) test. The CSF acid-fast staining was negative even after one week of incubation. Meningitis/encephalitis panel including *Cryptococcus*, varicella-zoster virus (VZV), *Streptococcus*, *Neisseria*, *Listeria*, human Parechovirus, human herpes virus 6, human simplex virus type 1 and 2, *Haemophilus influenzae*, *Escherichia coli* K1, Enterovirus, and CMV were also negative. Two days later, he started to have a rhythmic, stereotypical twitching of the right facial side involving eye blinking with generalized hypertonicity, tachycardia, cutaneous flushing, warmth, fever, and diaphoresis. The level of care was changed for the main suspicion of neuroleptic malignant syndrome secondary to a total of 35 mg of haloperidol. His mental status worsened and he was started on bromocriptine and dexmedetomidine infusion.

Although his focal twitching initially responded to benzodiazepines, it recurred with no electroencephalographic (EEG) correlation. EEG shows only rhythmic 1-3 Hz delta waves in the frontal area. Despite avoiding antipsychotic medication and treatment with bromocriptine, he had episodes of tachycardia, fever, and tachypnea that failed to respond to medications and promoted intubation. Persistent facial twitching that extended to the right lower limb and worsening clinical picture prompted another LP (lumbar puncture) to rule out paraneoplastic encephalitis and he was started on empiric high-dose steroids and plasmapheresis. In order to control his twitching, he was sequentially started on lacosamide, valproate, and levetiracetam but these were not effective. Ultrasound (US) of the scrotum was negative. The second LP and CSF were unremarkable and paraneoplastic serology including Anti Hu, Anti Yo, and Anti Ri antibodies were negative. However, the anti-NMDA receptor antibody was reactive in the CSF at 1:80 titer. No significant improvement was noted after pulse steroid and five sessions of plasmapheresis which prompted five doses of IVIG. Improvement in his ability to follow commands allowed for extubation but continued to have episodes of agitation, tachycardia, and flushing. A limited conversation was also possible.

Robotic thymectomy was performed. The pathological exam was consistent with thymic hyperplasia with no malignancy. More clinical improvement was noted afterward. Antiepileptic medications were gradually weaned off. No further episodes of agitation and sympathetic overactivity were noted.

## Discussion

Anti-NMDA receptor encephalitis may be the most common cause of autoimmune encephalitis. It has four stages including the prodromal phase, the psychotic phase, the unresponsive phase, and the hyperkinetic phase. Initially, it can be confused with acute psychosis which is believed to be secondary to reversible frontotemporal atrophy as this area has a high concentration of NMDA receptors [1]. Acute psychosis may be preceded by prodromal headache, flu-like symptoms, slurred speech, and cognitive dysfunction e.g memory concentration impairment. An important early clue for diagnosis is orofacial dyskinesia e.g., lip smacking or grimacing, and these can be mistaken for a seizure. Altered mental status and rigidity are common and can lead to, as in this case, diagnostic confusion with NMS (neuroleptic malignant syndrome). Other features can include autonomic dysfunction, hypoventilation, arrhythmia, and dissociative response e.g resisting to open the eyes but lacking a response to painful stimuli. In general, any acute atypical psychiatric symptoms or movement abnormality should raise suspicion and warrant serum and CSF serology.

A retrospective observational study of 252 patients found that 23.8% of the cases are associated with tumors and 6% of them were malignant. The most common is ovarian teratoma and other tumors can include small cell lung carcinomas, uterine adenocarcinoma, prostate adenocarcinoma, Hodgkin lymphoma, pineal dysgerminoma, neuroblastoma and pancreatic neuroendocrine tumor [2]. Around 80% of cases have no tumor [3]. The association with thymic hyperplasia is very rare and has been reported only a few times [4-6]. It was suggested in these cases that thymic tissue expression of NMDA receptors may contribute to immune response development, especially after exposure to triggers like herpes simplex infection.

In this case, two features of NMDA encephalitis led to confusion with NMS: rigidity and sympathetic overflow. Paroxysmal sympathetic hyperactivity (PSH) is commonly seen in acquired brain injury and is characterized by episodic tachycardia, hypertension, tachypnea, hyperpyrexia, diaphoresis, and abnormal motor posturing. A recent retrospective study of 24 patients with NMDA encephalitis found PSH in 50% of cases [7]. The prevalence was lower at 9% in a larger study involving 132 patients [8]. The most prominent features of PSH in this setting include tachycardia and hyperthermia. PSH may be the result of injury to autonomic regulatory areas including the insular cortex, anterior cingulate and ventral prefrontal areas, amygdala, hypothalamus, and spinal cord. PSH can affect the duration of hospitalization but not mortality and functional outcome [8].

## Conclusions

In conclusion, autoimmune encephalitis should always be suspected in unrelenting seizures and aggressive clinical course. NMDA encephalitis should be suspected in those with acute psychosis but no prior history or risk factors. Orofacial dyskinesia may occur and should not be confused with seizure especially if EEG is negative. Generalized rigidity may occur and should not be confused with NMS. Patients should be screened for malignancy as tumor removal provides a potential cure in these patients. Although rare, thymic hyperplasia can be associated with this syndrome and its removal helps in symptom control.
